# Innovative Bioscaffolds in Stem Cell and Regenerative Therapies for Corneal Pathologies

**DOI:** 10.3390/bioengineering11090859

**Published:** 2024-08-23

**Authors:** Federico Visalli, Federico Fava, Matteo Capobianco, Mutali Musa, Fabiana D’Esposito, Andrea Russo, Davide Scollo, Antonio Longo, Caterina Gagliano, Marco Zeppieri

**Affiliations:** 1 Department of Ophthalmology, University of Catania, 95123 Catania, Italy; federico.visalli13@gmail.com (F.V.);; 2 Department of Optometry, University of Benin, Benin City 300238, Edo State, Nigeria; 3 Department of Neurosciences, Reproductive Sciences and Dentistry, University of Naples Federico II, Via Pansini 5, 80131 Napoli, Italy; 4 Imperial College Ophthalmic Research Group (ICORG) Unit, Imperial College, 153-173 Marylebone Rd., London NW1 5QH, UK; 5 Eye Clinic Catania University San Marco Hospital, Viale Carlo Azeglio Ciampi, 95121 Catania, Italy; 6 Department of Medicine and Surgery, University of Enna “Kore”, Piazza dell’Università, 94100 Enna, Italy; 7 Department of Ophthalmology, University Hospital of Udine, p.le S. Maria della Misericordia 15, 33100 Udine, Italy

**Keywords:** scaffolds, corneal disease, stem cell, regenerative medicine, growth factors

## Abstract

Corneal diseases, which can result in substantial visual impairment and loss of vision, are an important worldwide health issue. The aim of this review was to investigate the novel application of bioscaffolds in stem cell and regenerative treatments for the treatment of corneal disorders. The current literature reports that organic and artificial substances create bioscaffolds that imitate the inherent structure of the cornea, facilitating the attachment, growth, and specialization of stem cells. Sophisticated methods such as electrospinning, 3D bioprinting, and surface modification have been reported to enhance the characteristics of the scaffold. These bioscaffolds have been shown to greatly improve the survival of stem cells and facilitate the regrowth of corneal tissue in both laboratory and live animal experiments. In addition, the incorporation of growth factors and bioactive compounds within the scaffolds can promote a favorable milieu for corneal regeneration. To summarize, the advancement of these groundbreaking bioscaffolds presents a hopeful treatment strategy for the regeneration of the cornea, which has the potential to enhance the results for individuals suffering from corneal disorders. This study highlights the possibility of utilizing the fields of biomaterials science and stem cell treatment to tackle medical demands that have not yet been satisfied in the field of ophthalmology.

## 1. Introduction

The cornea is a transparent structure that makes up the anterior portion of the fibrous tunic of the eyeball. It performs different functions. It consists of several layers, each of which performs specific functions. The epithelium is the most anterior and outermost corneal layer. This layer has several functions, including a protective barrier [1]. The Bowman’s layer is located below the epithelium. It is strong enough to allow protection of the underlying stroma [1]. The stroma represents the thickest layer of the cornea; it is mainly made up of collagen fibers, and it provides adequate structural support to the cornea [2]. The Dua’s layer is located between the corneal stroma and Descemet’s membrane. This layer has been recently discovered. It is an acellular layer. Its biomechanical properties are very important for corneal surgery [3]. Below the Dua layer is Descemet’s membrane; although it is a fairly thin layer, its structure allows it to support the underlying endothelium. Descemet’s membrane is also an important structure for corneal surgery [1]. The endothelium represents the innermost corneal layer; it performs several functions, including keeping the cornea dehydrated and transparent [1].

The cornea can be affected by different pathologies. Corneal infections derive from microorganisms such as bacteria, fungi, viruses, or parasites [4]. These can cause corneal ulcerations and subsequently lead to the formation of scars [5]. The corneal dystrophies represented by genetic diseases cause the abnormal accumulation of substrates in the cornea. Corneal degenerations are the result of age-related changes that result in thinning or dulling of the cornea. Corneal trauma can lead to the formation of scars with consequent loss of transparency. Dry eye syndrome is an important disease that affects more and more people. Parainflammation and chronic inflammation can create discomfort and damage to the ocular surface. Conventional treatments for corneal diseases, such as topical drugs, corneal transplants, and the use of artificial corneas, present several difficulties and limitations, such as the lack of available donors, the possible immune rejection of the transplanted flap, and other post-operative complications that can lead to transplant failure [6,7,8]. Despite the various advances made in these fields, the presence of these limitations makes it necessary to use new alternative therapeutic approaches. The use of stem cells seems to attract particular attention due to its potential to replace damaged corneal cells and promote tissue repair.

## 2. Bioscaffolds in Corneal Regeneration

Biological scaffolds are engineered to mimic the native extracellular matrix (ECM) of the cornea, providing a conducive environment for cell adhesion, proliferation, and differentiation. Recent advancements in scaffold design have significantly improved their efficacy [9]. The effectiveness of corneal bioscaffolds depends on several fundamental factors, which are necessary to ensure adequate tissue regeneration and repair of corneal tissues and possibly the restoration of visual function whenever possible [10,11,12,13]. First of all, the material used for the bioscaffold must be biocompatible in such a way as to avoid, as far as possible, adverse immunological and/or inflammatory reactions.

They must also promote cell adhesion and proliferation. Secondly, the bioscaffold must be optically transparent so as to guarantee the best possible visual quality and must mimic the cornea in its mechanical and architectural characteristics so as to act as a support for cell growth and maintain endocular pressures in the normal range. In the case of the cornea, the materials used should be transparent, or become transparent once implanted into the cornea, and have sufficient strength for suturing or gluing into place if used for full penetrating or anterior lamellar grafts. It is important that the transparency of the bioscaffold is maintained over time so as not to lose effectiveness. The bioscaffold must have controlled biodegradability so that it slowly reabsorbs over time and leaves room for regenerated native tissue [10]. Because the structures of the layers are distinct and have different functional requirements, it is unlikely that one biomaterial will be suitable for engineering the entire cornea [11]. Interestingly, synthetic polymers are rarely used as biomaterials in this application. As such, in the literature, many different naturally derived biomaterials have been investigated as potential scaffolds for corneal tissue engineering, with the most commonly used materials being amniotic membrane (AM), decellularized corneas, and collagen [11].

### 2.1. Materials for Bioscaffolds

Various materials have been used as corneal bioscaffolds, including collagen, gelatin, and chitosan, as well as synthetic materials such as polyurethanes and PEG, polyethylene glycol [13]. Whichever material is used, corneal bioscaffolds must necessarily have specific biophysical and biochemical characteristics, such as transparency, oxygen permeability, and intrinsic mechanical strength. With regard to materials, some studies have recently tried to use hybrid materials, i.e., natural and synthetic materials together, in order to create bioscaffolds with improved functional and biophysical properties [14,15]. It should also be mentioned that apart from the material used, a fundamental role is played by the manufacturing and processing techniques of the materials themselves, such as electrospinning and 3D printers [16].

#### 2.1.1. Natural Polymers

Natural polymers are often preferred for the production of bioscaffolds due to their excellent biocompatibility and biodegradability; natural polymers make excellent bioscaffolds. Among the most commonly used materials are collagen, chitosan, hyaluronic acid, gelatin, fibrin, alginine, and sericin. Tendentially, all these polymers can either be further chemically modified so as to improve certain mechanical properties and especially durability or used in a combined and hybrid manner in order to improve the properties of bioscaffolds and full integration with the host tissue.

(a) **Collagen**: Collagen is the structural protein most commonly represented in the cornea, so it has excellent biocompatibility, promotes cell adhesion and the production of extracellular matrix, and can be used in various formulations such as gels, sponges, or membranes [14]. Biological scaffolds are designed to mimic the normal extracellular matrix (ECM) of the cornea, providing an environment conducive to cell adhesion, proliferation, and differentiation. Collagen has been widely used to produce scaffolds as it is a material that allows excellent cell adhesion and has high biocompatibility. Several studies have shown that collagen-based scaffolds support the regeneration of different corneal layers, such as the corneal epithelial, stromal, and endothelial layers [14,17]. For example, Fagerholm P. et al. [18] have shown that human corneal equivalents created using collagen-based scaffolds are able to restore vision in animal models. In addition, it has been seen that decellularized porcine collagen scaffolds are able to integrate well with the host tissue, allowing high corneal transparency and high biomechanical resistance.

Collagen, however, has several limitations. Firstly, collagen exhibits relatively low mechanical strength and stiffness, making it unsuitable for applications that require significant structural support. This limit could be overcome by cross-linking collagen or combining it with other materials. However, structural weakness can be a major limitation for complex applications [1]. In addition, collagen is very susceptible to the enzymatic degradation of collagenases [2]. This leads to a rapid breakdown of the scaffolding, reducing stability and effectiveness over time [1]. Finally, it must be considered that pure collagen can show poor light transmission, a fundamental characteristic of corneal functions in which corneal transparency is a fundamental requirement. There are several cross-linking methods to improve these properties, such as EDC/NHS and riboflavin/UV light, but their use could introduce other problems related to long-term stability and biocompatibility [1,3].

(b) **Gelatin**: Gelatin, a hydrolyzed form derived from collagen, is used both for its excellent visco-elastic capabilities and for its ease of processing and handling. It is used in various forms (gels, films, sponges), alone or in combination, to improve the biomechanical properties of scaffolds. Its integration with bioactive peptides and cross-linking agents allows it to improve its mechanical properties and control degradation rates, making it suitable for different corneal applications. Research by Nosrati, H et al. [17] indicates that gelatin-based scaffolds functionalized with RGD peptides significantly improve cell adhesion and proliferation.

Gelatin, however, has a rapid degradation and does not have a high mechanical resistance alone. In particular, gelatin has a high degradation rate, which is a disadvantage in applications that require long-term stability. Rapid degradation would require chemical modifications that would allow its degradation rate to be controlled and, consequently, its mechanical properties improved. In addition, without a proper stabilization process, gelatin can dissolve under physiological conditions, leading to the loss of scaffolding integrity [3]. Despite possible modifications, the mechanical properties of gelatin may not be sufficient on their own for specific applications, thus requiring combination with other materials to increase its strength and durability in the long term [4]. Its poor mechanical stability under physiological conditions limits its use as an isolated material [3]. A final aspect to consider in gelatin-based compounds is thermosensitivity. The mechanical properties of gelatin are highly sensitive to temperature and environmental conditions, which can affect its clinical use. The melting point of gelatin is relatively low, making it unstable at body temperature [4].

(c) **Chitosan**: Chitosan is a chitin derivative with excellent antimicrobial properties and good biocompatibility. It is used on its own or in combination with other polymers, has the ability to form biofilms, gels, and fibers, and is easily manipulated and processed [19].

Chitosan, however, has limited mechanical strength and can be difficult to process uniformly. The main limitations of the use of chitosan concern solubility problems. In fact, chitosan can be difficult to dissolve and work under physiological conditions, requiring specific solvents or pH adjustments. Its solubility is highly dependent on pH and the degree of deacetylation, which makes processing and application very complex and significantly increases the costs of its production [5]. In addition, chitosan exhibits moderate mechanical strength, which may not be adequate for all corneal applications without further modification or reinforcement [5]. Chitosan has limited mechanical strength and can be difficult to process uniformly.

The main limitations of the use of chitosan concern solubility problems [6]. In fact, chitosan can be difficult to dissolve and work under physiological conditions, requiring specific solvents or pH adjustments [6]. Its solubility is highly dependent on pH and the degree of deacetylation, which makes processing and application very complex and significantly increases the costs of its production [5]. In addition, chitosan exhibits moderate mechanical strength, which may not be adequate for all corneal applications without further modification or reinforcement. Finally, although chitosan is biodegradable, its rate of degradation can be highly variable and needs adjustments to achieve controlled and uniform degradation [6]. The rate of degradation can also be influenced by the degree of deacetylation and the presence of other modifying agents.

(d) **Hyaluronic Acid**: Hyaluronic acid, a key element of the extracellular matrix, contributes massively to corneal hydration and lubrication due to its strong water retention capacity; it allows the support of cell proliferation and migration. It is usually used as a hydrogel for corneal scaffolds in order to create a suitable environment for tissue regeneration [13].

Pure hyaluronic acid alone, however, has low mechanical strength and typically needs to be used in combination with other materials to provide adequate structural support. Because of this characteristic, its use could be limited to applications where high mechanical integrity is not required [2]. In addition, hyaluronic acid hydrogels can be too soft for load-bearing applications [3]. Another aspect to consider is the rapid degradation of pure hyaluronic acid. This can be a problem in long-term applications. Cross-linking allows its degradation to be slowed down but can affect biocompatibility, mechanical properties, and production costs [2].

#### 2.1.2. Synthetic Polymers

Synthetic polymers are widely used for the fabrication of corneal bioscaffolds due to their versatility and the possibility of modifying their physio-chemical characteristics, making the treatment as customized as possible. Among the most commonly used synthetic polymers are polyethylene glycol (PEG), polyvinyl alcohol (PVA), polyurethanes, polyacid lactic acid (PLA), polyglycol (PGA), polydimethylsiloxane (PDMS), poly(ε-caprolactone) (PCL), and poly(lactic-co-glycolic acid) (PLGA) [16,17,18,19,20]. These offer adjustable properties for scaffold design.

(a) **Polylactic Acid (PLA)**: A biodegradable polymer with adjustable mechanical properties. PLA, a biodegradable polymer derived from lactic acid, and PGA are often used together to create rapidly biodegradable scaffolds with a rapid tissue response and good mechanical properties that promote tissue regeneration [14]. However, the main limitations of the use of PLA relate to its degradation products. In fact, its degradation can produce acidic byproducts capable of causing local inflammation, with a consequent negative impact on surrounding tissues. The formation of an acidic environment is an obstacle to cell growth and tissue regeneration [2].

(b) **Polycaprolactone (PCL)**: PCL is a biodegradable polymer with good biocompatibility and slow degradation, which is why it is mainly used in all those cases where a durable scaffold is required. Furthermore, it can be easily machined to form microstructures. The main limitation to its use could concern cellular attachment. PCL has a lower cell attachment rate than natural polymers, often requiring surface modification to improve bioactivity and ensure effective cell integration. Its hydrophobic nature can limit cell adhesion and proliferation [4].

(c) **Polyethylene Glycol (PEG)**: PEG is a hydrophilic polymer with excellent biocompatibility, easy to modify chemically and mechanically, has the ability to form hydrogels, and is non-toxic. In addition, by modifying porosity and other physio-chemical characteristics, it promotes both corneal cell migration and proliferation, making it an excellent bioscaffold [10]. PEG lacks mechanical strength, and for this reason, it must be combined with other materials that increase its mechanical strength and support necessary for corneal applications, as it can be too flexible and weak on its own. Pure PEG hydrogels may not possess the mechanical integrity required for corneal scaffolds [3]. Another aspect to consider is that PEG lacks intrinsic bioactivity, which means that it does not promote cell adhesion or proliferation on its own. For this, it often needs functionalization or combination with bioactive molecules to improve cellular interactions [7].

(d) **PLGA**: A copolymer of PLA and PGA that possesses hybrid characteristics between the two materials mentioned above. All these materials can be further chemically modified to incorporate drugs, antimicrobial agents, growth factors, and other substances that promote tissue regeneration. Furthermore, depending on the fabrication technique, such as electrospinning and the use of 3D printers, there is a high degree of customization to create scaffolds tailored to the patient [10,15,19].

The comparative properties of different scaffold materials are listed in Table 1.

## 3. Fabrication Techniques

Different bioscaffold fabrication techniques are available to replicate the complex corneal microenvironment. In particular, there are different techniques for the creation of bioscaffolds that allow the support of the growth of stem cells and the regeneration of tissues [20,21,22,23,24,25,26,27,28,29,30,31]. The main manufacturing techniques used to create bioscaffolds are electrospinning, 3D bioprinting, hydrogel formulations, and decellularization.

### 3.1. Electrospinning

Electrospinning is a technique that allows the production of scaffolds. The scaffolds that are formed appear to mimic the structure of ECM and are able to promote cell adhesion and proliferation. For the realization of electrospinning, the formation of a high-voltage electric field is necessary. This electric field is applied to a polymer solution, resulting in the formation of continuous fibers that are collected on a target substrate [22]. The generated scaffolds have a high porosity and surface area; these are important characteristics for nutrient diffusion and cell infiltration. By adjusting different parameters, it is possible to control the diameter and alignment of the fibers (such as the concentration of the polymer and the strength of the electric field generated) [23]. This is a very advantageous aspect because it allows the creation of scaffolds tailored to specific needs. A recent study has shown that the use of electrospun nanofibers enhanced with bioactive molecules such as growth factors allows the significant improvement of tissue regeneration [2]. In particular, electrospun scaffolds loaded with bioactive proteins showed a greater adhesion and proliferation capacity of corneal stem cells. Another study showed that electrospun scaffolds loaded with proteins such as collagen and gelatin improve cell adhesion and proliferation, promoting corneal tissue regeneration [8]. Finally, the incorporation of nanoparticles into the electrospun fibers provides antimicrobial properties, improving the safety and effectiveness of the scaffold [9].

### 3.2. Three-Dimensional Bioprinting

Three-dimensional bioprinting is a technique that is based on the deposition, layer by layer, of bioinks (mixtures of cells and biomaterials) that allows the construction of three-dimensional scaffolds with specific structural characteristics [24]. This technique allows the spatial arrangement of different types of cells within a single scaffold, trying to mimic the complex corneal structure [25]. In corneal tissue engineering, 3D bioprinting is capable of creating scaffolds with controlled porosity, mechanical properties, and biocompatibility [26]. It also facilitates the incorporation of different growth factors and other bioactive molecules, thereby improving cell proliferation and differentiation. Recent studies have investigated the use of patient cells to create personalized scaffolds, thereby reducing the risk of immune rejection [10]. In particular, one study reported that 3D-printed scaffolds loaded with mesenchymal stem cells allow for better corneal regeneration in animal models [11]. Another study used bioprinting to create a multilayer corneal structure, trying to mimic the normal architecture of the cornea. Layer-by-layer assembly allows precise control over growth factor release kinetics, ensuring sustained therapeutic effects and minimizing cytotoxicity [2]. Finally, the development of new advanced bioprinting techniques will allow the fabrication of complex corneal scaffolds capable of addressing the specific needs of the individual patient.

### 3.3. Hydrogel Formulation

Hydrogel formulations create highly hydrated substrates that allow for the encapsulation and delivery of different cell types. In situ gelation is a technique that is based on the formation of hydrogels directly within the target site. This technique also makes it possible to provide a supporting scaffold for cell growth. Hydrogels can be formed from natural or synthetic polymers that undergo gelation through physical or chemical cross-linking [27]. It would appear that hydrogels can be injected directly into the corneal defect, thus forming a strong scaffold. Within these hydrogels, it is possible to incorporate molecules and bioactive cells capable of promoting tissue regeneration and integration with the host tissue, thus reducing possible rejection [11]. A recent study used hydrogels loaded with stem cells and growth factors to treat corneal lesions [12]. This study showed significant regeneration of corneal tissue and a reduced risk of immune rejection. Another study showed that hydrogels based on natural polymers, such as hyaluronic acid and collagen, provide excellent biocompatibility and are able to support the growth of corneal cells.

### 3.4. Decellularization

Decellularization uses native corneal tissues that have been treated to remove cellular components, leaving only an ECM scaffold. Decellularization involves the removal of different cellular components from donor tissues while preserving the structural and biochemical properties of ECM [28]. This reduces immunogenicity and improves biocompatibility. This process is capable of producing a natural scaffold that provides an ideal microenvironment for cell attachment, migration, and differentiation. The decellularized corneal matrices can subsequently be repopulated with stem cells derived from the patient himself, thus minimizing the risk of possible immune rejection. Techniques for decellularization include different methods, which can be chemical, enzymatic, and physical, each capable of effectively removing cells while maintaining the integrity of the ECM [28]. Chemicals such as sodium dodecyl sulfate (SDS) and Triton X-100 are commonly used. However, their harsh nature compromises the integrity of the CME. Recent studies have focused on optimizing the concentration and period of exposure to try to balance cell removal and ECM preservation. Studies have shown that optimized SDS protocols are able to maintain the mechanical properties and transparency of decellularized corneal scaffolds [13]. Enzymatically decellularized porcine corneas have shown to retain key components of the ECM. These key components, such as collagen and glycosaminoglycans, are essential for cell adhesion and functions. Some recent studies have tried to optimize decellularization methods [14]. Optimized protocols using sodium dodecyl sulfate (SDS) have been shown to maintain the mechanical properties and transparency of decellularized corneal scaffolds [15]. Enzymatically decellularized scaffolds also retain key components of the ECM, essential for adhesion and cellular functions, such as collagen and glycosaminoglycans. In addition, the use of decellularized matrices from different sources, including human and animal tissues, has shown promise in creating effective corneal scaffolds.

### 3.5. Nanofabrication

Nanofabrication allows for the creation of nanoscale structures, thereby improving cell–scaffold interactions and nutrient diffusion. In addition, collagen processing techniques exploit the high biocompatibility and structural properties of collagen to produce scaffolds capable of supporting the proliferation and differentiation of corneal cells. Some techniques, such as microfabrication in soft lithography and solvent casting with particle leaching, allow the creation of scaffolds with controlled porosity; this feature is important for driving cell behavior and differentiation [22,25]. These different fabrication techniques, which are often used in combination with each other, enable the development of complex bioscaffolds that can effectively support stem cell-based corneal regeneration, thus addressing the limitations of current therapeutic approaches [2]. The use of advanced materials in nanofabrication, such as graphene and its derivatives, has made it possible to improve some of the scaffold’s properties, such as stability. Finally, the combination of nanofabrication with other production techniques, such as electrospinning and bioprinting, would allow the creation of multifunctional scaffolds with high mechanical properties and biological performance.

The advantages and limitations of these techniques are summarized in Table 2 [8,9,10,11,12,13,14,15,16,17,18,19,20,21,22,23,24,25,26,27,28,29,30,31]. Table 3 lists the main studies regarding bioscaffolds for corneal regeneration [17,18,19,20,21,22,23,24,25,26,27,28].

## 4. Functional Strategies

Different functionalization strategies are fundamental for the proper functioning of bioscaffolds as they allow for better scaffold–cell interactions, promote specific cellular responses, and improve overall tissue integration. Key strategies include surface modification, incorporation of growth factors, and integration of nanoparticles.

### 4.1. Surface Modifications

There are different methods of modifying the surface of bioscaffolds, such as chemical modification, physical treatments, and biological coatings. Chemical modification typically involves grafting bioactive molecules onto the surface of the scaffold [21]. For example, RGD (arginine–glycine–aspartic acid) peptides, derived from the ECM’s fibronectin protein, are used to improve cell adhesion [22]. Several studies have shown that the incorporation of RGD peptides into scaffolds significantly improves corneal epithelial cell adhesion and proliferation compared to scaffolds that have not been incorporated with RGD peptides. For example, Hsu, C.C. et al. [6] showed that collagen scaffolds functionalized with epidermal growth factor (EGF) were able to significantly modify the proliferation and migration of epithelial cells [23]. In addition, cellular interactions can be improved by altering the surface energy of scaffolds and their hydrophilicity through physical treatments such as plasma treatment and UV irradiation. For example, it has been seen that plasma treatment would appear to improve cell adhesion and growth by improving the adsorption capacity of PCL scaffolds [13]. Biological coatings coat the scaffold of the bioscaffold with ECM proteins or other bioactive substances. Collagen coatings promote the adhesion and differentiation of limbal stem cells, which are crucial for corneal epithelial regeneration [24].

### 4.2. Incorporation of Growth Factors

The incorporation of growth factors into the scaffold promotes cell differentiation and tissue regeneration. Growth factors, in particular, are essential signaling molecules that regulate cell proliferation, differentiation, and migration. The incorporation of growth factors into bioscaffolds allows the creation of an ideal microenvironment for tissue regeneration [22]. However, the rapid degradation and short half-life of growth factors represent one of the main challenges to their use. To this end, slow-release systems such as growth factor encapsulation or incorporation of growth factors within the scaffold matrix have been developed. For example, to promote angiogenesis and cell proliferation, which are important features in corneal repair, VEGF (vascular endothelial growth factor) and bFGF (basic fibroblast growth factor) have been incorporated into hydrogel scaffolds [15]. In addition, the possible creation of a gradient of growth factors within the bioscaffolds allows the creation of natural signaling gradients present in the tissues. This approach has been used to guide the differentiation of stem cells into specific types of corneal cells [25]. For example, it would seem that the presence of an EGF (epidermal growth factor) gradient within a collagen bioscaffold improves the migration and proliferation of corneal epithelial cells [26].

### 4.3. Integration of Nanoparticles

Nanoparticles can be integrated into bioscaffolds to deliver drugs, growth factors, or genetic material in a controlled manner [27,28,29,30,31,32,33,34]. This integration can enhance the scaffold’s regenerative properties and provide additional therapeutic benefits. Nanoparticles could be used to deliver anti-inflammatory or antifibrotic drugs directly to the site of corneal injury, thereby reducing complications and improving healing rates. Gene delivery via nanoparticles could be used to introduce specific genes that promote cell survival, proliferation, or differentiation [28,29,30,31,32,33,34]. Nanoparticles such as silver or gold nanoparticles would appear to have inherent antibacterial properties and, therefore, could be integrated into bioscaffolds to prevent infections, which are a common complication in corneal lesions [29]. In a study by Moradi, S. et al. (2020) [35]., it would seem that scaffolds coated with silver nanoparticles significantly reduce bacterial colonization by supporting the growth of corneal cells. Incorporating growth factors and drugs into scaffolds requires careful design to ensure controlled release rather than burst release. Burst release can lead to high local concentrations that may be cytotoxic, whereas controlled release provides a sustained therapeutic effect [25].

These innovations demonstrate how the implementation of targeted functionalization strategies can not only improve the properties of bioscaffolds but also open new frontiers in corneal regenerative medicine, offering more effective and personalized solutions for patients with corneal pathologies.

## 5. Integration of Stem Cells and Bioscaffolds

Stem cells, in particular induced pluripotent stem cells (iPSCs) and mesenchymal stem cells (MSCs), have the characteristic of possessing great potential for corneal regeneration due to their ability to differentiate into various types of corneal cells [30,31,32,33,34].

### 5.1. Limbal Stem Cells (LSC)

Limbal stem cells (LSCs) are essential for the maintenance of the corneal epithelium. Bioscaffolds could be used to expand and deliver LSCs to the damaged corneal surface, thereby promoting regeneration and preventing scarring [31]. In a study by Mahmood, N et al. [32], collagen scaffolds seeded with LSCs were transplanted onto the corneal surface of rabbits with limbal stem cell deficiency. The results would appear to show significant epithelial regeneration and reduced increased scarring compared to controls, demonstrating the potential advantage of LSC-loaded scaffolds in corneal repair [8]. In a clinical study by Zhou, J et al [33], limbal stem cell transplantation using fibrin-based scaffolds was evaluated in patients who had a limbal stem cell deficiency. The study reported a 76% success rate in restoring a stable and transparent corneal epithelium over a 12-month follow-up period [33]. Limbal stem cells (LSCs) appear to hold great promise in restoring the corneal epithelium. Recent studies show better regeneration and healing of scars when administered via collagen or fibrin-based scaffolds.

### 5.2. Mesenchymal Stem Cells (MSCs)

Mesenchymal stem cells (MSCs) can differentiate into corneal stromal cells and endothelial cells. These characteristics make them especially suitable for the treatment of deeper corneal layers. Bioscaffolds loaded with MSCs appear to have potential in stromal defect repair and endothelial regeneration [8]. Mesenchymal stem cells (MSCs), with their ability to differentiate into stromal and endothelial cells, appear to be suitable for repairing deeper corneal layers. Hydrogels and electrospun nanofibers are commonly used to deliver MSCs to the corneal stroma. In a study by Vattulainen, M. et al [34] a hyaluronic acid-based hydrogel loaded with MSCs was used to try to treat corneal stromal defects in a rabbit model [34]. The hydrogel–MSC construct significantly improved stromal regeneration and transparency compared to untreated controls. Currently, there are few clinical trials directly assessing MSC-loaded bioscaffolds for corneal regeneration. MSCs have immunomodulatory properties and secrete trophic factors that promote tissue repair. Moradi, S et al. [35] reported that MSCs delivered via collagen scaffolds significantly improved wound healing and reduced scarring in a model of corneal alkali burn [35]. However, the safety and efficacy of MSC therapy in other applications provide a strong rationale for further clinical investigation in corneal pathologies [36].

### 5.3. Induced Pluripotent Stem Cells (iPSCs)

Induced pluripotent stem cells (iPSCs) are able to generate specific cells and offer the advantage of personalized therapy. Bioscaffolds are able to support the differentiation of iPSCs into corneal epithelial, stromal, and endothelial cells. Three-dimensional bioprinting techniques allow the creation of very complex scaffold structures that guide the differentiation and integration of iPSCs into corneal tissue. A study by Wang, M et al [8] demonstrated the use of iPSC-derived corneal endothelial cells on a decellularized corneal scaffold in a rabbit model. The transplanted constructs showed successful integration, improving corneal transparency and endothelial function. iPSCs can be differentiated into corneal, stromal, and endothelial epithelial cells. A study by Wang, M et al. [8] appears to show that iPSC-derived corneal epithelial cells restored corneal transparency and integrity in a rabbit model of limbal stem cell deficiency [8]. This approach offers an unlimited cell source, overcoming the limitations of donor-derived cells.

## 6. Challenges and Future Directions

Despite all this progress, several challenges still remain. In fact, there should be guarantees on the integration and long-term functionality of regenerated tissues, the minimization of immune responses, and the development of scalable production methods for clinical applications. Future research should focus on:

### 6.1. Immunogenicity and Biocompatibility

Biocompatibility and immunogenicity are key aspects for a corneal bioscaffold to be effective in performing its function. Any immune system response to the bioscaffold may compromise its integrity and long-term functionality, preventing the proliferation and differentiation of corneal cells. Similarly, biocompatibility must be carefully assessed; the use of materials that are non-toxic to cells and tissues is a prerequisite for creating a favorable environment for cell proliferation [37]. As far as immunogenicity is concerned, we are helped by the fact that keratoplasties are generally considered immuno-privileged interventions because there are no blood and lymph vessels; however, a rejection of the donated cornea or bioscaffold is still possible. In order to further reduce the risk of rejection [38], it is possible to use some special techniques, e.g., the use of decellularized and decalcified bioscaffolds of animal origin (e.g., tilapia and other fish), the use of A. mylitta-derived fibroin or similar silk fibroin films seeded with endothelial cells, or the use of special synthetic polymers such as sericin. Advanced materials and surface modification techniques are being explored to enhance scaffold compatibility with host tissues. For example, surface functionalization with bioactive peptides can improve cell adhesion and reduce immune responses [39].

### 6.2. Scaffold Design and Customization

Design and customization are a crucial moment in the realization of corneal bioscaffolds. Through biometric and biomimetic studies, it is possible to create bioscaffolds that are as similar as possible to the native corneal tissue, with mechanical and biochemical characteristics that are durable and functional. Nowadays, thanks to innovative approaches, such as the use of electrospun nanofibers, incorporation of growth factors, and extracellular matrix components, bioscaffolds are increasingly high-performance. Moreover, thanks to advances in manufacturing processes, such as bioprinting using 3D printers, it is possible to create structures that are customized to the patient’s characteristics, which is a great advantage both in reducing immune rejection and in improving functional adaptation. Three-dimensional bioprinting would seem to be a promising approach to obtaining customized scaffolds capable of adapting to the specific needs of individual patients. In particular, 3D bioprinting could create scaffolds with precise microarchitectures that mimic native corneal structures, improving cell alignment and function [39,40].

### 6.3. Integration with Advanced Technologies

Integrating bioscaffolds with advanced technologies such as gene editing, tissue engineering, stem cell therapy, and bioinformatics can improve their functionality and therapeutic potential [41]. The use of advanced techniques, such as the decellularization of donor corneas to create a naturally bioactive extracellular matrix or the use of nanofibrous nanomaterials created by electrospinning, can significantly improve these parameters [29]. For example, CRISPR-Cas9 technology can be used to modify stem cells to improve regenerative abilities. Combining iPSCs with decellularized scaffolds could provide both structural support and a source of regenerative cells [29]. There are also hybrid and innovative techniques, such as the use of compressed collagen as a dense support structure in combination with a laser-perforated electrospun matrix.

## 7. Applications for Corneal Bioscaffolds

### 7.1. Corneal Neovascularization

Corneal neovascularization is a condition that can significantly impair vision. Bioscaffolds capable of releasing anti-angiogenic agents, such as anti-VEGF (vascular endothelial growth factor), could represent a new therapeutic solution for this condition. According to a study, the use of scaffolds based on collagen and hyaluronic acid impregnated with anti-VEGF allows the reduction of not only vascular invasion but also corneal inflammation in some animal models [26]. Another study confirmed that the use of scaffolds allows the control of inflammation and vascular growth, improving corneal homeostasis and preserving transparency [27].

The use of bioscaffolds is particularly promising in chronic inflammatory ocular diseases in which traditional therapies do not allow a complete resolution of the clinical picture. In addition, bioscaffolds would enable targeted and sustained delivery of therapeutic agents while supporting the regeneration and healing of corneal tissues. The ability of bioscaffolds to deliver drugs in a controlled manner minimizes systemic exposure and subsequent side effects, improving the overall safety and efficacy of treatment [28]. In fact, bioscaffolds that release anti-angiogenic agents, such as VEGF inhibitors, which are essential for preventing the formation of new blood vessels, have made it possible to act directly on the formation of these vessels. Bioscaffolds may also incorporate multiple therapeutic agents to address different aspects of the pathogenesis of corneal neovascularization.

### 7.2. Keratoconus

Keratoconus is a progressive disease that causes thinning and cone-shaped deformation of the cornea. Bioscaffolds, especially those based on cross-linked collagen, are theoretically able to stabilize the corneal structure. Scaffolds have all the characteristics to offer a less invasive alternative to corneal transplantation, especially in the early stages of the disease [30]. In addition, bioscaffolds can provide structural support to the weakened cornea in keratoconus. Scaffolds are able to integrate with the corneal stroma, providing greater mechanical stability and potentially slowing or stopping the progression of the disease [30].

Some studies support this possible approach by demonstrating how bioscaffolds help strengthen corneal structure and facilitate tissue regeneration, thereby improving corneal thickness and reducing conical deformation of the cornea [31]. In addition, the ability of bioscaffolds to release cross-linking agents or growth factors in a localized and sustained manner could also be exploited. This method could improve the results of procedures such as corneal collagen cross-linking (CXL) by providing a more controlled and prolonged therapeutic effect over time. Studies highlight the potential of combining bioscaffolds with iontophoresis-assisted CXL to improve riboflavin-assisted penetration and distribution, resulting in improved corneal strengthening [31,32]. Finally, by providing physical support, scaffolds allow corneal shape and integrity to be maintained, which is crucial for patients with keratoconus to avoid worsening corneal ectasia. Clinical trials are essential to make the most of the great potential of bioscaffolds in the management of keratoconus.

### 7.3. Corneal Epithelial Disorders

Corneal epithelial disorders, including limbal stem cell deficiency (LSCD), represent a significant challenge in ophthalmology due to their impact on vision, the limited availability of effective treatments, and, more generally, the quality of life of patients with these diseases. Limbal stem cell deficiency (LSCD) is a condition that can lead to severe epithelial problems, impairing corneal epithelial regeneration [33]. Bioscaffolds could offer promising solutions for treating these conditions by providing structural support and facilitating drug delivery in a precise manner. Bioscaffolds could serve as a support for limbal stem cell culture and transplantation.

A study published in Stem Cell Research & Therapy showed that the use of fibrin scaffolds loaded with limbal stem cells significantly improved epithelial regeneration and visual function in preclinical models of LSCD [33,34]. Scaffolds are not only able to support cell regeneration but also improve the stability and function of the ocular surface. Several studies have shown that bioscaffolds are able to integrate effectively with corneal tissue, thus promoting the regeneration of the corneal epithelium and restoring its barrier function [35]. Bioscaffolds can be engineered to support and facilitate the survival and proliferation of LSCs so as to ensure their proximity to the limbus [35]. This would allow for better reconstruction of the corneal epithelium and prevent conditions such as conjunctivalization, in which the corneal surface is covered with conjunctival cells [35]. In addition, bioscaffolds, combined with LSCs or progenitor cells, are able to restore limbal niches, thus supporting the regeneration of a healthy and functional corneal epithelium [36]. Clinical and preclinical studies have demonstrated the potential of this approach to restore vision and improve corneal transparency in patients with LSCD.

### 7.4. Corneal Ulcers

Corneal ulcers and wounds pose significant clinical challenges because of their potential to cause severe vision damage and complications if not treated effectively. Bioscaffolds could hold promise for improving the treatment and healing of these corneal lesions by providing structural support, promoting cell regeneration, and providing therapeutic agents [37]. Bioscaffolds are not only capable of supporting tissue regeneration but also play a crucial role in modulating the wound healing process. In addition, due to their ability to incorporate growth factors such as epidermal growth factor (EGF) and fibroblast growth factor (FGF), they are able to accelerate epithelial healing and reduce the risk of scarring [36].

Bioscaffolds allow corneal transparency to be maintained by preventing scar formation, controlling the inflammatory response, and promoting the formation of healthy tissues. One of the key benefits of bioscaffolds is the ability to deliver drugs directly to the site of injury. Scaffolds can be impregnated with antibiotics, antifungals, and anti-inflammatory drugs, ensuring a prolonged and localized release. This targeted delivery helps to effectively manage infections and reduce inflammation, which are critical factors for the success of therapy. For example, scaffolds loaded with vancomycin or ciprofloxacin have shown greater efficacy in treating bacterial keratitis while maintaining high local concentrations of the drugs [26]. This is particularly important in the management of keratitis caused by multidrug-resistant pathogens. Prolonged release of drugs directly at the site of infection ensures greater efficacy of therapy. In addition, by ensuring localized administration, bioscaffolds reduce the need for frequent administration and thus help reduce the development of drug resistance.

### 7.5. Corneal Endothelial Dysfunction

Corneal endothelial dysfunction results from conditions such as Fuchs’ endothelial dystrophy, surgical trauma, or congenital hereditary endothelial dystrophy, and these conditions result in impaired corneal hydration and transparency. Bioscaffolds can be used to transplant endothelial cells derived from induced pluripotent stem cells (iPSCs). Studies have shown that scaffolds with PLGA are able to support the growth and differentiation of endothelial cells [20]. Bioscaffolds could, therefore, represent a valid alternative to traditional treatments, such as corneal transplants, as they would be able to provide an ideal substrate for the growth and maintenance of corneal endothelial cells [26]. In addition, bioscaffolds could also be used to deliver cultured human corneal endothelial cells (HCECs) or stem cell-derived endothelial cells directly to the corneal area affected by the defect. This method would allow the replacement of dysfunctional endothelial cells with healthy and functional cells.

### 7.6. Corneal Transplantation

Corneal transplantation is often the last step in serious corneal diseases that do not respond to other treatments. Due to the global shortage of donor corneas and the risk of rejection of the transplanted flap, bioscaffolds have emerged as a promising alternative, offering structural support and improving the integration and function of transplanted tissues. Bioscaffolds could improve transplant outcomes as they are able to provide structural support and promote tissue regeneration. In addition, scaffolds containing corneal stem cells may be able to support epithelium and stroma regeneration, thereby improving the transparency and function of the transplanted cornea [38].

Decellularized bioscaffolds, from which cellular components have been removed, reduce the risk of immune rejection by minimizing the presence of foreign antigens. These scaffolds are able to maintain the natural architecture and biochemical properties of the ECM, facilitating tissue regeneration and minimizing inflammatory responses [26]. This approach is fundamental as it allows the improvement of the success rates of corneal transplants. Bioscaffolds can also be designed to deliver therapeutic agents, such as anti-inflammatory drugs or growth factors, directly to the transplant site [39]. For example, scaffolds loaded with anti-inflammatory agents would reduce post-operative inflammation and improve healing times. Instead, the combination of bioscaffolds with stem cells, such as MSCs or iPSCs, would offer a regenerative approach to corneal transplantation [40]. These cells can differentiate into different types of corneal cells, providing a renewable source for repairing damaged tissues.

## 8. Regulatory and Ethical Considerations

Bringing bioscaffolding therapies from the lab to the patient’s bedside requires addressing substantial regulatory and ethical challenges. To ensure that these therapies can be safely and effectively integrated into clinical practice, it is crucial to overcome numerous regulatory hurdles and standardize manufacturing processes [42]. In addition, it is necessary to demonstrate the safety and efficacy of these new therapies through rigorous preclinical and clinical tests, together with the establishment of specific guidelines (World Health Organization (WHO)). Regulatory frameworks need to evolve to keep pace with advances in technology. It is, therefore, necessary to develop specific protocols for the experimentation and production of bioscaffolds [25].

The World Health Organization (WHO) emphasizes the importance of developing comprehensive and specific guidelines that address the unique challenges posed by advanced therapy medicines [25]. Ethical considerations are equally important and critical. Transparency in research and development is essential to build trust among the population. Of fundamental importance is the formation and signing of the informed consent of the patient and family members, especially when it comes to new therapies that may involve unknown and unpredictable risks. Ethics review committees and regulatory bodies must work together to assess potential risks and benefits, ensuring that patient safety is prioritized at all stages of clinical trials [25].

It is essential to address the issue of equitable access to care. Access disparities can result from high costs, limited availability, and complexity of procedures. Implementing public policies that support equitable access and distribution of therapies is critical in ensuring that all patients who could benefit from these innovations have the opportunity to do so. Large-scale clinical trials are needed to validate the safety and efficacy of bioscaffold therapies in human patients [26]. These studies must be meticulously designed to meet regulatory standards and ethical requirements, thereby ensuring reliable results. In addition, continuous patient monitoring and long-term follow-up studies would be important to understand the full impact of these therapies on patients’ health and to identify any long-term risks [25]. Finally, international collaboration and knowledge sharing make it possible to accelerate the use of bioscaffolds in clinical practice. International organizations, academic institutions, and industry must work together to develop global standards and promote collaborative research. [43,44].

## 9. Conclusions

The use of biological scaffolds and stem cells in regenerative therapies would seem to offer a promising alternative to traditional therapies [45,46]. These new and innovative therapies would have potential benefits for millions of corneal disease patients worldwide. Personalized medicine would require the design of customizable scaffolds that can meet the needs of individual patients. [47] Techniques such as 3D bioprinting would seem to provide tailor-made solutions in this regard [48]. However, the reproducibility of these custom scaffolds, moving from the lab to the patient’s bedside, remains a challenge that requires further innovation and optimization of existing resources [49].

Ongoing research seeks to address these challenges by trying to develop multifunctional and intelligent scaffolds that can dynamically interact with the host environment. The integration of reactive materials capable of changing properties in response to specific stimuli (e.g., pH, temperature) is an exciting area of exploration that is still little explored [50]. Finally, the possible combination of bioscaffolds with other emerging technologies, such as CRISPR-based gene editing and advanced imaging techniques, would seem to be a very promising avenue for the future of innovative therapies for corneal diseases [51].

## Figures and Tables

**Table 1:** Comparative properties of scaffold materials.

Material	Biocompatibility	Mechanical Strength	Transparency	Degradation Rate
Collagen	High	Moderate	High	Moderate
Gelatin	High	Moderate	High	High
Chitosan	Moderate	High	Moderate	Moderate
Hyaluronic Acid	High	Low	High	High
Fibrin	High	Moderate	High	High
Silk Fibroin	High	High	Moderate	Moderate
Alginate	High	Low	Moderate	High
PCL	High	High	Moderate	Low
PEG	High	Low	High	High
PGA	High	High	Low	High
PLGA	High	High	Moderate	High

**Table 2:** Advantages and limitations of fabrication techniques.

Technique	Advantages	Limitations
**Electrospinning**	Mimics ECM structure [29,31]	Difficulty in producing thick, 3D scaffolds [29,31]
	High surface area and porosity, enhances cell adhesion/proliferation [29,31]	Limited mechanical strength [30,32]
	Incorporates bioactive molecules, antimicrobial properties [8,30]	Potential for bead formation affecting uniformity [8]
**3D Bioprinting**	Precise cell/biomaterial arrangement, mimics natural tissue [30,31]	High cost and complexity [30,31]
	Creates multilayered structures, multiple cell types [30,31]	Limited resolution for fine structures [29]
	Incorporates growth factors/bioactive molecules [29]	Slow printing speed [29]
**Hydrogel**	High water content, supports cell encapsulation/delivery [8,30]	Poor mechanical properties [30,31]
	Flexible design with natural/synthetic polymers [30]	Rapid degradation rates [31]
	Injectable, forms robust scaffold in situ [8,29]	Swelling/contraction affecting stability [29]
**Decellularization**	Produces natural ECM scaffolds, reduced immunogenicity [29,30]	Variability in tissue quality [29,30]
	Maintains key ECM components for cell adhesion/function [30]	Risk of incomplete cell removal [31]
	Potential for repopulation with patient-derived cells [8,30]	Complex, time-consuming process [30]
**Nanofabrication**	Nanoscale structures enhance cell interactions/nutrient diffusion [31]	High cost, technical complexity [30]
	Techniques like soft lithography, solvent casting for controlled porosity [8,31]	Potential for contaminants during fabrication [31]
	Advanced materials like graphene enhance properties [8]	Limited by material availability and specialized equipment [8]

**Table 3:** Summary of key studies on bioscaffolds for corneal regeneration.

Study	Scaffold Type	Functionalization Methods	Cell Type(s) Used	Key Findings
Fagerholm P. et al. [18]	Collagen-based	None	Human corneal epithelial cells	Restored vision in animal models, good integration with host tissue
Nosrati H. et al. [17]	Gelatin-based	RGD peptides	Corneal epithelial cells	Improved cell adhesion and proliferation
Fagerholm P. et al. [18]	Decellularized porcine	Optimized SDS protocol	None	Maintained mechanical properties and transparency of decellularized corneal scaffolds
Tayebi, T et al. [19]	Decellularized porcine	Enzymatic (trypsin and dispase)	None	Retained critical ECM components essential for cell attachment and function
Yan, B et al. [21]	Collagen-based	None	MSCs	Improved wound healing and reduced scarring in a model of corneal alkali burn
Yu, X et al. [28]	Collagen-based	Combined chemical and enzymatic approach	None	Improved decellularization, retaining critical ECM components for tissue engineering
Teimouri, R et al. [23]	Poly(ethylene glycol)-based	None	Human corneal endothelial cells	Biodegradable and biocompatible hydrogel films for regeneration of corneal endothelium
Fagerholm P. et al. [18]	Amniotic membrane-based	None	Keratocytes	A novel tissue-engineered corneal stromal equivalent
Ahearne, M. et al. [10]	Silk fibroin-based	None	Human corneal endothelial cells	Human corneal endothelial cell growth on a silk fibroin membrane
Tayebi et al. [19]	Chitosan-based	None	Various cell types	Supports cell attachment, proliferation, and bone regeneration

## Data Availability

Data are contained within the article.
